# DEPTOR promotes survival of cervical squamous cell carcinoma cells and its silencing induces apoptosis through downregulating PI3K/AKT and by up-regulating p38 MAP kinase

**DOI:** 10.18632/oncotarget.8131

**Published:** 2016-03-16

**Authors:** Kalanghad Puthankalam Srinivas, Remadevi Viji, Vipin Mohan Dan, Indira Sukumaran Sajitha, Rajappan Prakash, Puthan Valappil Rahul, Thankayyan R. Santhoshkumar, Subhadra Lakshmi, Madhavan Radhakrishna Pillai

**Affiliations:** ^1^ Cancer Research Program, Rajiv Gandhi Centre for Biotechnology, Thiruvananthapuram-695014, Kerala, India; ^2^ Division of Cancer Research, Regional Cancer Centre, Thiruvananthapuram-695011, Kerala, India

**Keywords:** DEPTOR, cervical cancer, PI3K/AKT, ERK and p38, apoptosis

## Abstract

DEPTOR is an endogenous inhibitor of mTOR complexes, de-regulated in cancers. The present study reveals a vital role for DEPTOR in survival of cervical squamous cell carcinoma (SCC). DEPTOR was found to be overexpressed in both cervical SCC cells and tissues and it's silencing in cervical SCC cells induced apoptosis, mainly by up-regulation of p38 MAPK and by inhibiting PI3K/AKT pathway via a feed-back inhibition from mTORC1-S6K. DEPTOR silencing resulted in reduced expression of the nitric oxide synthases iNOS and eNOS, as well as increased activation of ERK1/2 and p38 MAP kinases. Activation of AKT signaling by overexpression of constitutively active-AKT (CA-AKT) failed to overcome the apoptosis caused by DEPTOR silencing. Similarly pharmacological inhibition of ERK also failed to control apoptosis. However pharmacological inhibition of p38 MAPK rescued the cells from apoptosis, indicating the major role of p38 MAPK in cell death induced by DEPTOR silencing. DEPTOR was also found to regulate ERK1/2 in an AKT dependent manner. DEPTOR knockdown induced cell death in SiHa cells overexpressing the anti-apoptotic Bcl-2 and Bcl-xL, indicating strong survival role of DEPTOR in these cells. DEPTOR overexpression activated PI3K/AKT by relieving the negative feed-back inhibition from mTORC1-S6K. DEPTOR regulation was also observed to be independent of HPV E6/E7 oncoproteins, but it might be a molecular co-factor contributing to cervical carcinogenesis. In summary, DEPTOR is found to promote survival of cervical SCC cells and its reduction induced apoptosis via differential effects on PI3K/AKT and p38 MAPK and can be a potential target in cervical SCC.

## INTRODUCTION

The mammalian target of rapamycin (mTOR) is an evolutionarily conserved serine/threonine kinase integrating intracellular and extracellular signals. It serves as a master regulator of several metabolic processes including growth, proliferation, survival and autophagy. The pathways controlled by these complexes are frequently de-regulated in cancers [1-3]. DEPTOR is identified as an endogenous *in vivo* inhibitor of mTOR, binds to both mTORC1 and mTORC2 and inhibits their activities [4]. By blocking mTOR activity, DEPTOR in general should act as a tumor suppressor [5]. Its overexpression was known to induce apoptosis in pancreatic cancer cells and its loss of expression was thought to contribute to pancreatic tumorigenesis [6]. However, high levels of DEPTOR was reported to be essential for the survival of various cancer cells [4, 7, 8]. Thus, DEPTOR expression has frequently been reported to be essential for the survival and proliferation of tumor cells in multiple myeloma, thyroid cancer, paclitaxel resistant ovarian cancer and hepatocellular carcinoma [4, 7, 9-11].

Cervical cancer is the fourth most common cancer among women worldwide (Globocan, IARC, 2014). High-risk Human papillomaviruses account for almost all cervical carcinomas [12, 13]. p53 and pRb are known to be degraded by HPV E6 and E7 and are best described host cellular targets of HPV E6 and E7 oncoproteins [14]. High-risk HPV E6 is also known to bind with several PDZ domain containing cellular proteins such as CBP/p300, BARD1, c-MYC, E6-BP/ERC 55, E6TPI, ORF-3, Mcm 7, Paxillin, hD1g, MAGI-1, MUPP-1, hScrib and NHERF1 [15, 16]. HPV E6 is reported to activate PI3K/AKT/mTOR complex [15, 17]. Reports also indicate HPV E7 expression activates AKT [18, 19]. We hypothesized a possible interaction/regulation between DEPTOR and HPV oncoproteins E6/E7, as DEPTOR is an endogenous *in vivo* inhibitor of mTOR complexes. Peterson et al., [4] reported that DEPTOR silencing in HeLa (adenocarcinoma derived cell line) resulted in increased cell proliferation. To study the regulation of DEPTOR by HPV oncoproteins, we initially assessed the effects of DEPTOR silencing in cervical cancer cell lines SiHa, ME-180 (Both squamous cell carcinoma derived) and also in HeLa. DEPTOR silencing indeed increased the cell proliferation in HeLa cells. Surprisingly, DEPTOR silencing induced cell death in SiHa and ME-180 cells. In this study, we detected overexpression of DEPTOR in cervical SCC primary cancer tissues and also report mechanistic evaluation of DEPTOR in cell survival and cell death processes and also the differential regulation of DEPTOR in cervical squamous cell carcinoma (SCC) and adenocarcinoma (AC) cells.

## RESULTS

### DEPTOR silencing induces apoptosis in cervical squamous cell carcinoma cells

**Figure 1:**
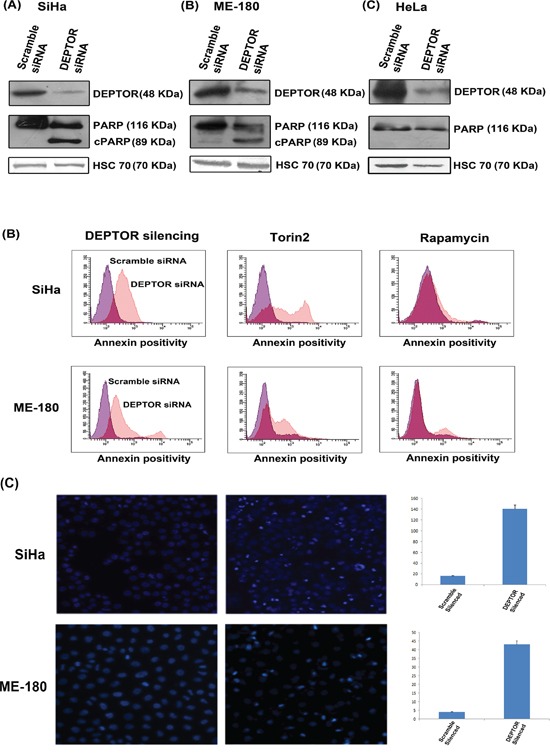
DEPTOR knockdown induces apoptosis in cervical SCC cells **A.** DEPTOR silencing induced apoptosis in SiHa and ME-180 cells, as evident by PARP cleavage in both cells. DEPTOR silencing in cervical AC derived cell HeLa induces cell proliferation, evident by absence of PARP cleavage. **B.** Annexin binding assay analyzed by FACS indicates that DEPTOR silenced SiHa and ME-180 population showed more than ten-fold annexin positivity than the control population. Torin2, a strong dual mTOR inhibitor, and rapamycin treated cells showed approximately four- and two-folds of annexin positive population in comparison to their respective DMSO treated controls. **C.** DEPTOR silencing induced nuclear condensation in both SiHa and ME-180 cells. The nuclei of the DEPTOR silenced respective cells are condensed with bright appearance in comparison with scrambled control cells.

To address the role of DEPTOR in cervical cancer cells, we knocked down DEPTOR in SiHa, ME-180 and HeLa cells (Figure 1A). DEPTOR silencing in HeLa cells induced proliferation, and no cell death was observed, as reported earlier [4]. However, quite interesting results were observed in DEPTOR silenced cervical cancer cells SiHa and ME-180, with significant apoptotic cell death after 48 hours of DEPTOR silencing, as evident by PARP cleavage (Figure 1A) and from annexin binding assay (Figure 1B). In annexin binding assay for quantification of apoptosis by FACS, the DEPTOR-silenced SiHa and ME-180 cells showed approximately ten-fold annexin positive population when compared to the scramble siRNA transfected cells (Figure 1B) and this is far stronger in comparison to the cells treated with reported mTOR inhibitors rapamycin and Torin2 (Figure 1B). Nuclear condensation, a general aspect of apoptosis was also analyzed in SiHa and ME-180 cells using fluorescent microscopy and the DEPTOR silenced cells showed relatively high percentage of nuclear condensation in comparison to the respective controls (Figure 1C). The colony formation assay also suggests the inability of DEPTOR silenced SiHa cells to form colonies in comparison to the control silenced cells, indicating the cell death under DEPTOR silencing conditions (Supplementary Figure 2). All these data substantiate that DEPTOR silencing induces significant cell death in cervical SCC cells, but not in AC cells. Several studies have previously reported differential gene expression between AC and SCC of the uterine cervix [20-22].

### DEPTOR silencing induces caspase dependent apoptosis

**Figure 2:**
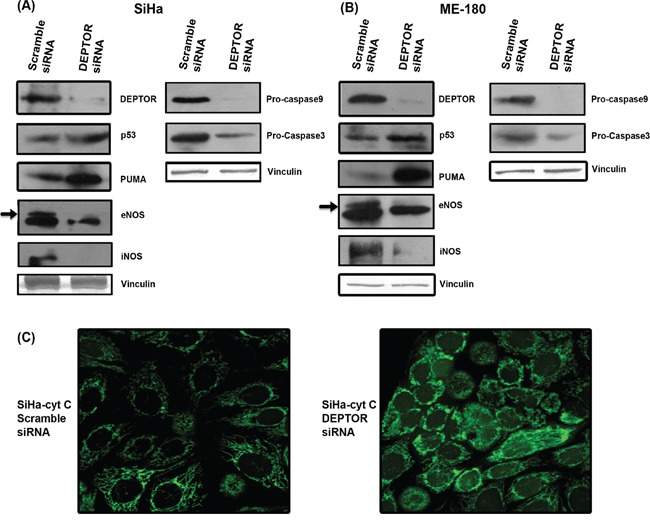
DEPTOR silencing up-regulates p53 and PUMA, down-regulates NOS enzymes and induce caspase dependent apoptosis **A** and **B.** DEPTOR silencing induced PUMA, a strong pro-apoptotic protein in a p53 dependent manner in both SCC cells. Marked inhibition of both iNOS and eNOS in DEPTOR silenced cells indicate the pro-survival nature of nitric oxide in these cells. Induction of p53 and PUMA, and also inhibition of NOS enzymes may have contributed to the caspase dependent apoptosis, evident by reduction in procaspases9 and 3. **C.** Confocal image showing cytochrome c release, an event in caspase dependent apoptosis in SiHa cells overexpressing cyt c-GFP.

Nuclear condensation, a characteristic aspect of apoptosis, is executed by the cleavage of structural proteins of nucleus by activated caspases. Both intrinsic and extrinsic pathways contribute to activation of caspases. The intrinsic apoptotic pathway is characterized by permeabilization of the mitochondrial membrane and release of Cyt C into cytoplasm, which forms a multi-protein complex apoptosome that initiates activation of the Caspase cascade [23, 24]. To analyze the Caspase-dependent apoptosis induced by DEPTOR silencing in cervical cancer cells, we assessed levels of Caspase-9 and Caspase-3 by immunoblotting. In DEPTOR-silenced SiHa and ME-180 cells, there is a clear reduction in the levels of Procaspase-9 and Procaspase-3 (Figure 2A and 2B) in comparison to the control silenced cells. Similarly, Cyt C release due to permeabilization of mitochondria was also analyzed in SiHa cells stably expressing Cyt C-EGFP [25]. DEPTOR silencing caused release of Cyt C from mitochondria to the cytoplasm (Figure 2C). All these data provide evidence for Caspase-dependent cell death through intrinsic pathway.

### Induction of p53, PUMA and suppression of nitric oxide synthases in DEPTOR silenced cells

Since the systems used were HPV-transformed cervical SCC cells, we assessed levels of p53 and pRb proteins that are normally degraded by HPV E6 and E7. Ubiquitinous degradation of p53 and pRb by high risk HPV E6 and E7 are well known molecular events in cervical carcinogenesis [26]. Induction of p53 levels were significant in both DEPTOR-silenced SiHa and ME-180 cells (Figure 2A and 2B), while the difference in levels of pRb was not clearly evident (data not shown). The tumor suppressor p53 induces numerous genes that play an important role in promotion of apoptosis [27]. PUMA (p53 up-regulated mediator of apoptosis) is one such transcriptional target of p53 and binds to BCl_2_ family proteins, causing mitochondrial membrane permeabilization and induces apoptosis via Cyt C/Apaf1-dependent mechanism [27]. Immunoblotting of DEPTOR-silenced SiHa and ME-180 cells revealed marked increase in the levels of PUMA (Figure 2A and 2B). Inhibition of PI3K/AKT pathway increases activity of p53 and induces apoptosis [28]. Thus, reduced AKT activity could account for the elevated p53 and PUMA proteins and associated effects on apoptosis.

The role of NO is also highly varied in cancer and several studies contradicting its role in progression of cancer had been reported [29]. Strong expression of iNOS and eNOS were found in breast cancers, intestinal cancers and other cancers [29]. The PI3K/AKT pathway is known to directly regulate NOS and production of NO [30, 31]. PI3K-AKT was well known to regulate NO production in endothelial cells by regulating eNOS [32]. Consistent with this, we observed that marked inhibition in the levels of NOS enzymes iNOS and eNOS was observed in DEPTOR silenced SCC cells (Figure 2A and 2B), indicating that nitric oxide (NO) might play an important role in the survival of cervical cancer cells.

### DEPTOR knockdown have distinct effects on mTORC1 and mTORC2 and inhibits PI3K/AKT pathway

**Figure 3:**
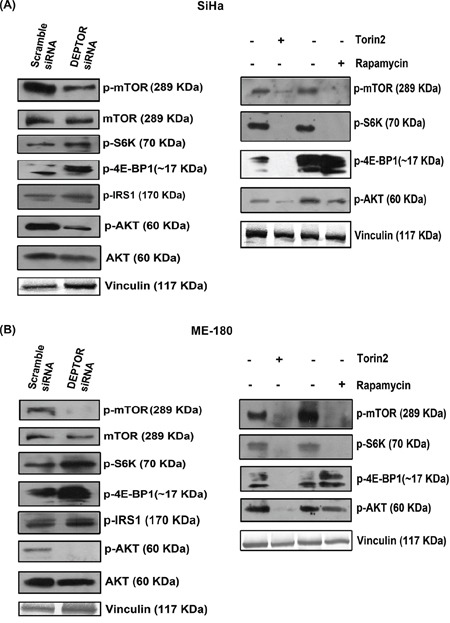
DEPTOR silencing inhibited the PI3K-AKT pathway **A** and **B.** DEPTOR silencing inhibited PI3K/AKT pathway via feed-back inhibition from mTORC1 and S6K in both SCC cells, as evident by activity of mTORC1, characterized by phosho-S6K and 4E-BP1 and by inhibition of PI3K/AKT, characterized by up-regulation of phopsho-IRS1 and inhibition of AKT levels. The mechanism is compared with the status of mTORC1 and mTORC2 activities in cells treated with rapamycin and Torin2.

To assess the mechanistic pathways responsible for apoptotic cell death induced by DEPTOR silencing, we examined mTORC1 and mTORC2 pathway status [4]. Initial experiments focused on the phosphorylation status of mTORC1 and mTORC2 substrates under DEPTOR silencing conditions. In DEPTOR-silenced SiHa and ME-180 cells, phosphorylated forms of S6K (Thr 389) and 4E-BP1 (Thr 37/46) increased (Figure 3A and 3B), when compared to the scramble silenced controls. This indicates increased mTORC1 and S6K activity. The phospho-mTOR at Ser 2448 was also down regulated in both DEPTOR silenced cells (Figure 3A and 3B). The phosphorylation of mTOR at Ser 2448 is mediated by AKT. However mTOR can activate itself by auto phosphorylation at Ser 2481, which activates S6K and 4E-BP1 [4, 33].

PI3K-AKT pathway is the central survival signaling, often de-regulated in cancers. Sustained activation of AKT was known to contribute to the pathogenesis of several cancers and also pathological angiogenesis [34-36]. In the present study, DEPTOR-silencing also resulted in the suppression of phosphorylated forms of AKT (Ser 473) (Figure 3A and 3B), indicating suppression of mTORC2 activity, which is in contrast to what was reported in HeLa, where DEPTOR silencing activated AKT [4]. DEPTOR silencing also resulted in up-regulation of phospho-IRS1 levels in both cells (Figure 3A and 3B). This regulation indicates at the strong feed-back inhibition of IRS1-PI3K-AKT pathway from S6K. IRS1 is known to contain several PI3K binding domains and these are responsible for the activation of PI3K, which further activates AKT and S6K phosphorylates IRS1 to prevent its binding to PI3K [37-41]. In contrast, rapamycin inhibited phospho-S6K and had no effect on phosphorylated forms of 4E-BP1 (Thr 37/46) or on the phospo-AKT (Ser 473), while Torin2 inhibited phosphorylated forms of S6K (Thr 389), 4E-BP1 (Thr 37/46) and AKT (Ser 473), indicating inhibition of both mTOR complexes (Figure 3A and 3B). Rapamycin was known to inhibit S6K but not 4E-BP1 (Figure 3A and 3B). All these data indicate that cell death in cervical SCC cells by DEPTOR silencing is mainly due to inhibition of PI3K/AKT pathway.

### Ectopic overexpression of DEPTOR activates PI3K/AKT

**Figure 4:**
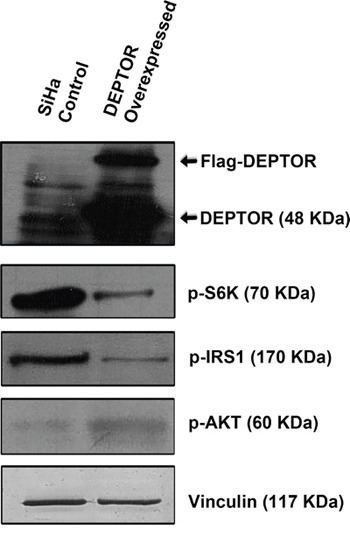
DEPTOR overexpression activates PI3K/AKT by relieving negative feed-back inhibition from S6K

Ectopic overexpression of Flag-DEPTOR in SiHa cells activated the PI3K/AKT pathway by relieving the negative feed-back inhibition from mTORC1 and S6K. DEPTOR overexpression inhibited the activity of mTORC1 as defined by the activity of its downstream target S6K (Figure 4). The down-regulation of S6K activity relieves the negative feed-back on to the IRS1/PI3K/AKT, as characterized by the down-regulation of p-S6K and p-IRS1 levels in DEPTOR overexpressed cells (Figure 4). S6K phosphorylates IRS1 to prevent its binding to PI3K and further preventing the activity of PI3K/AKT [40, 41]. DEPTOR overexpression resulted in the activation of AKT, as defined by the increased phosphorylation of AKT (Ser 473) (Figure 4), suggesting the activation of AKT by relieving the negative feed-back inhibition from mTORC1/S6K under conditions of DEPTOR overexpression [4, 37-39].

### Over expression of CA-AKT could not completely overcome apoptosis caused by DEPTOR silencing and DEPTOR was found to regulate ERK in AKT dependent manner

**Figure 5:**
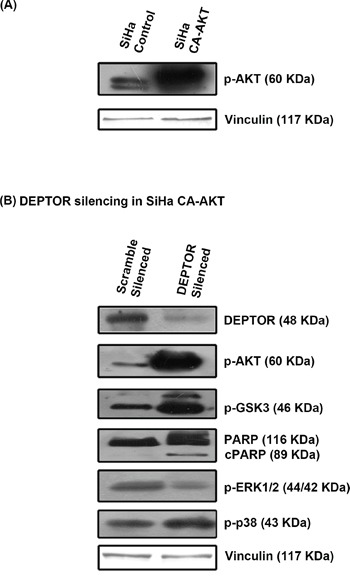
CA-AKT overexpression failed to overcome apoptosis induced by DEPTOR silencing **A.** Status of constitutively active AKT (CA-AKT) in SiHa **B.** CA-AKT failed to prevent apoptosis in DEPTOR silenced SiHa cells, as evident by PARP cleavage and DEPTOR was found to regulate ERK in an AKT dependent manner.

Since PI3K/AKT pathway is primarily inhibited by DEPTOR silencing and the molecules p53 and NOS are regulated through AKT pathway, we overexpressed a catalytically/constitutively active AKT (CA-AKT) to see if this would overcome apoptosis caused by DEPTOR silencing (Figure 5A and 5B). Overexpression of CA-AKT reduced apoptosis caused by DEPTOR silencing, but could not completely stop the process of cell death, as evident by PARP cleavage (Figure 5B). CA-AKT overexpression rescued down-regulation of AKT pathway, as evident by the levels of AKT and its downstream target GSK3β (Figure 5B), observed to be highly inhibited by the negative feed-back inhibition from S6K imparted by DEPTOR silencing. This suggested that the apoptosis of cervical cancer cells induced by DEPTOR silencing is not only mediated through the inhibition of PI3K/AKT pathway. The up-regulated forms of ERK1/2 and p38 MAPK under DEPTOR silencing conditions might also be contributing to apoptosis.

The activity status of ERK1/2 and p38 MAP kinases was also analyzed under DEPTOR silencing conditions in SiHa cells overexpressing CA-AKT. The phosphorylated forms of ERK1/2 decreased in cells over expressing CA-AKT under DEPTOR silencing conditions (Figure 5B), which is in contrast to the silencing in cells without CA-AKT. While levels of p38 MAPK seems to be unaffected by CA-AKT over expression, as no significant difference was observed under DEPTOR silenced conditions in cells with or without CA-AKT over expression (Figure 5B). The activation of ERK1/2 under DEPTOR silencing conditions seems to be a mechanism of cross inhibition between ERK and AKT pathways [42]. This data indicates that DEPTOR regulates ERK1/2 in an AKT dependent manner.

### Up-regulation of ERK and p38 MAP kinases

**Figure 6:**
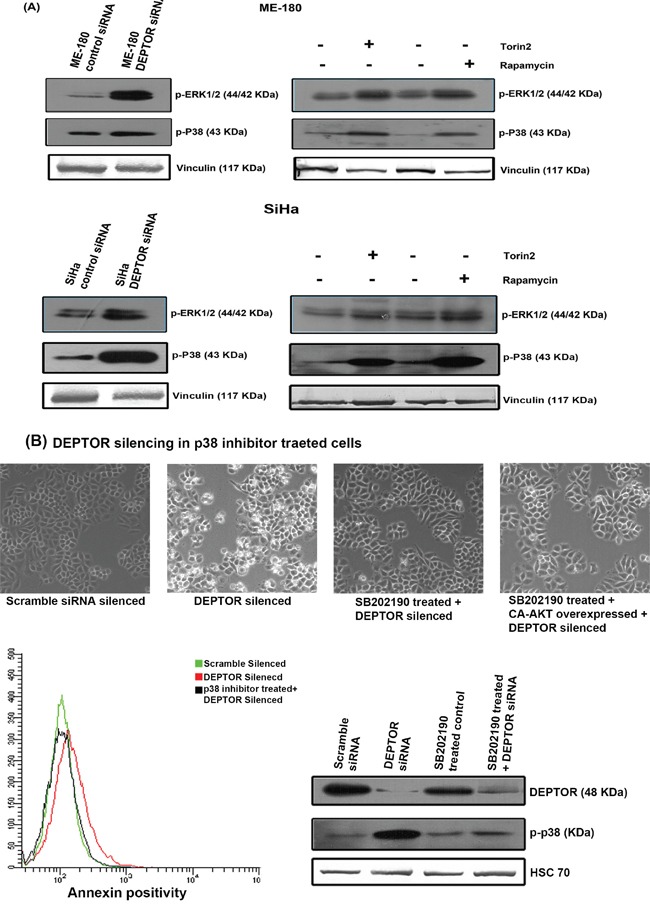
Up-regulation of ERK1/2 and p38 MAP kinases by DEPTOR silencing in cervical SCC cells and Pharmocological inhibition of p38 MAPK attenuates cell death due to DEPTOR silencing **A.** DEPTOR silencing in SCC cells induced increased levels of both activated ERK1/2 and p38 MAPK, indicating a complex regulation of DEPTOR in various vital cell signaling pathways. The cells treated with rapamycin and Torin2 also showed similar induction of both ERK1/2 and p38 MAP kinases **B.** Inhibition of p38 MAPK activity attenuates the apoptosis induced by DEPTOR silencing.

As we observed inhibition of AKT pathway, we next analyzed the expression levels of ERK and p38 MAP kinases under DEPTOR silencing conditions. ERK signaling pathways are reported to be involved in cell proliferation, differentiation, cytoskeleton reorganization and cell migration. Moreover ERK's are also reported to be involved in cellular stress and apoptosis [43, 44]. p38 MAPK has also been shown to be involved in a variety of cellular processes, including cell survival and death processes [43, 45]. Marked increase in the phosphorylated forms of both ERK1/2 (Thr202/Tyr204) and p38 MAPK (Thr180/Tyr182) in DEPTOR-silenced SiHa and ME-180 cells was evident in comparison to the control cells (Figure 6A). Similarly, increased levels of these two were also noticed in rapamycin and Torin2 treated cells (Figure 6A). The up-regulated forms of ERK1/2 and p38 MAPK might have also contributed to the apoptosis of cells under DEPTOR silencing. In our study, direct interaction between endogenous DEPTOR and ERK1/2 was not observed (data not shown), however the clear activation of ERK and p38 MAPK by DEPTOR silencing suggests a positive input by mTORC1 activation. This could occur through suppression of PI3K/AKT, which has been observed to suppress ERK.

The effect of ERK up-regulation on apoptosis mediated by DEPTOR silencing was studied by pharmacological inhibition of ERK1/2 using PD98059 inhibitor. The SiHa cells were pretreated for 3 hours with 10μM PD98059 before DEPTOR silencing, along with proper control. The treatment of cells with ERK inhibitor could not overcome the apoptosis caused by DEPTOR silencing, as indicated by annexin binding assay using FACS. The annexin binding assay suggested the percentage of annexin positivity in cells treated with ERK inhibitor coupled with DEPTOR silencing is almost similar to the cells with DEPTOR silencing alone (Supplementary Figure 3), ruling out the role of ERK in DEPTOR silencing mediated apoptosis. The status of ERK activity under the condition of DEPTOR silencing, pre-treatment with ERK inhibitor coupled with DEPTOR silencing and pre-treatment with ERK inhibitor coupled CA-AKT overexpression and DEPTOR silencing was also shown (Supplementary Figure 3).

### Up-regulated p38 MAPK activity is responsible for apoptosis mediated by DEPTOR silencing

The role of p38 MAP kinases in apoptosis is reasonably well documented. Several reports suggest the role of p38 MAPK in promoting apoptosis [46, 47]. As pharmacological inhibition of ERK could not overcome the apoptosis caused by DEPTOR silencing, we concentrated on the role of p38 MAP kinase. The effect of p38 MAPK up-regulation on apoptosis mediated by DEPTOR silencing was studied by pharmacological inhibition of p38 MAPK using SB202190 inhibitor. The SiHa cells were pretreated for 3 hours with 10μM SB202190 before DEPTOR silencing, along with proper control. The treatment of cells with p38 MAPK inhibitor indeed overcome the apoptosis caused by DEPTOR silencing, as indicated by annexin binding assay using FACS (Figure 6B). The annexin binding assay suggested the percentage of annexin positivity in cells treated with p38 MAPK inhibitor coupled with DEPTOR silencing is almost similar to the control cells with scramble siRNA silencing (Figure 6B), while the DEPTOR silenced cells showed approximately 7-10 times the annexin positivity in comparison to the control and p38 MAPK inhibitor pretreatment coupled with DEPTOR silencing. The microscopic images also support this identification (Figure 6B). The status of blots for DEPTOR and phospho-p38 MAPK under conditions including pre-treatment with p38 MAPK inhibitor, DEPTOR silencing and pre-treatment with p38 MAPK inhibitor coupled with DEPTOR silencing were also shown (Figure 6B). This data clearly indicates the role of p38 MAPK in promoting cell death caused by DEPTOR silencing.

### DEPTOR regulation is independent of HPV E6/E7 oncoproteins

**Figure 7:**
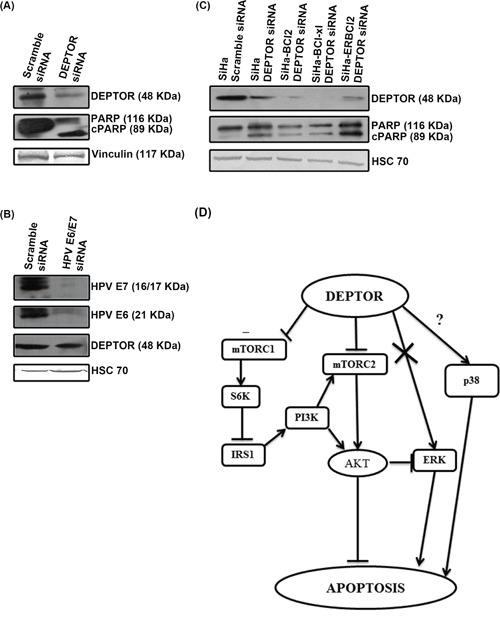
DEPTOR expression is independent of HPV oncoproteins E6/E7 and DEPTOR silencing can bypass anti-apoptotic effect of Bcl2 and Bcl-xL **A.** DEPTOR silencing induced apoptosis in HPV negative cervical cancer cell C33A. **B.** HPV E6/E7 silencing has no effect on DEPTOR expression. **C.** DEPTOR silencing also bypasses the anti-apoptotic regulation of Bcl2 and Bcl-xL, as evident by PARP cleavage in SiHa cells overexpressing Bcl2, ER-Bcl2 and Bcl-xL under DEPTOR silencing conditions. **D.** Schematic representation of DEPTOR induced signaling in cervical SCC.

High-risk HPV oncoproteins E6 and E7 are known to ubiquitously degrade several host cellular proteins. HPV E6 contains a PDZ domain that is known to interact with PDZ domain of several host cellular proteins [16] and high risk HPV E6 is known to activate the mTOR complex [15]. DEPTOR interacts with mTOR via its PDZ domain and also contains two DEP domains, whose functions are still unknown [4]. In general, DEP domains are reported to mediate protein-protein interactions. HPV E6 is reported to interact with several host cellular proteins via its PDZ domain and alter their activities [12, 13]. We initially hypothesized that HPV E6 might bind to DEPTOR and therefore checked for a possible interaction between DEPTOR and HPV E6 as well as with E7 by immunoprecipitation assays. Our repeated immunoprecipitation assays failed to detect any direct interactions between DEPTOR and HPV E6/E7 (data not shown). Next, we analyzed whether DEPTOR silencing can induce apoptosis in HPV negative cervical cancer cell line C33A. DEPTOR silencing indeed resulted in the apoptotic cell death of C33A cells, as evident by PARP cleavage (Figure 7A). To support this, we also silenced E6 and E7 in SiHa cells using HPV E6/E7 siRNA and checked the status of DEPTOR. No reduction in the levels of DEPTOR was observed with E6/E7 silencing (Figure 7B). All these data indicate a probable HPV E6/E7-independent mechanism of DEPTOR regulation in cervical SCC cells.

### DEPTOR knockdown induced apoptosis in SiHa cells overexpressing Bcl-2, Bcl-xL and ER targeted Bcl-2

Bcl-2 family proteins are evolutionarily related proteins regulating apoptosis. These proteins are localized to mitochondria and govern mitochondrial outer membrane permeabilization. These can be pro-apoptotic (such as Bax, Bok, Bak and BAD) and anti-apoptotic (such as Bcl_2_, Bcl-xL and Bcl-W). Pro-apoptotic Bcl_2_ proteins promote release of apoptogenic factors (such as Cyt C, SMAC) to initiate and execute apoptosis, while the anti-apoptotic Bcl_2_ proteins inhibit release of Cyt C by preventing the formation of mitochondrial apoptosis-induced channel (MAC) [48]. To investigate whether silencing DEPTOR can induce cell death in cells overexpressing anti-apoptotic molecules Bcl-2 and Bcl-xL, we used SiHa cells overexpressing Bcl_2_, Bcl-xL and ER targeted Bcl-2, previously reported from our laboratories [25]. DEPTOR silencing indeed induced apoptosis in SiHa cells overexpressing Bcl-2, Bcl-xL and ER-Bcl-2, as evident by PARP cleavage (Figure 7C). These data indicates that DEPTOR silencing can bypass the anti-apoptotic effect of Bcl-2 and Bcl-xL to induce apoptosis in cervical cancer cells overexpressing these strong anti-apoptotic molecules.

### Primary cervical tumors show strong DEPTOR overexpression

**Figure 8:**
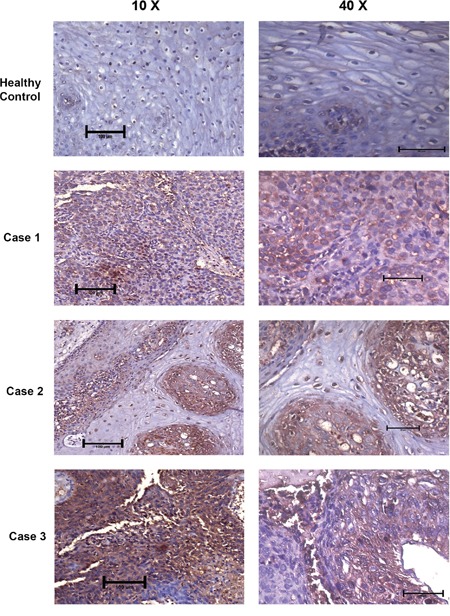
DEPTOR is overexpressed in human cervical squamous cell carcinoma tissues DEPTOR overexpression was clearly evident in all the three human cervical SCC tissues tested. Strong DEPTOR expression is visible in case 3 followed by case 1 and case 2 in comparison with the apparent healthy cervix tissue, where DEPTOR expression is very feeble. Koilocytes, a hollow zone around the nucleus, characteristic of HPV infection is also clearly visible (Case 2) with DEPTOR expression in nucleus. DEPTOR is predominantly expressed in both cytoplasm and nucleus.

DEPTOR overexpression was reported in hepatocellular carcinoma tissues from HCC patients and its expression was linked to the poor survivability of HCC patients [10]. DEPTOR was also found to be overexpressed in thyroid carcinoma cells and tissues, and thyroid carcinoma patients showing high DEPTOR expression were found to be susceptible to earlier recurrence and poorer survival chances [9] and overexpression of DEPTOR was also found to correlate with the poor survival rate in patients with multiple myeloma [4, 49]. We also analyzed primary cervical SCC human tissue specimens for evaluation of DEPTOR expression by immunohistochemistry. Results clearly indicate the overexpression of DEPTOR, in comparison to normal cervical tissue where DEPTOR expression was found to be minimal, localized in the cytoplasm (Figure 8; Supplementary Figure 4). Koilocytes, characteristic of HPV-associated cervical cancers were also observed in these tumor sections. Earlier, it was thought that DEPTOR is localized to cytoplasm, but the immunohistochemical analyses of the primary tumors revealed nuclear and membrane localization of DEPTOR, along with the cytoplasmic localization (Figure 8; Supplementary Figure 4), the role of which still needs to be studied.

## DISCUSSION

DEPTOR is an endogenous *in vivo* inhibitor of mTORC1 and mTORC2 [4, 50]. As mTOR activation is reported to be the hall mark of several cancers, DEPTOR in general should act as tumor suppressor and its expression was reported to have growth suppression effects in pancreatic cancer cells [4, 6]. However, DEPTOR was also reported to have an oncogenic role and found to be overexpressed in many cancers such as multiple myeloma, paclitaxel resistant ovarian cancer, hepatocellular carcinoma and thyroid cancer [3, 4, 6, 8]. We started the present study with an aim to address the possible interaction/regulation between DEPTOR and HPV E6/E7, since HPV E6/E7 are known to interact with several host cellular proteins [12, 13]. We have also further tried to decipher the role of DEPTOR in cervical cancer cells and address its role in cell survival and cell death processes. Previously, it was shown that DEPTOR silencing in HeLa promoted cell survival and proliferation [4], and we also observed a similar effect in our studies (Figure 1A). However, we observed that the effect of DEPTOR silencing was quite different in the SCC cell lines SiHa and ME-180, as DEPTOR silencing induced strong apoptosis in SiHa and ME-180 cells (Figure 1A and 1B). Several studies have previously reported differential gene expression between AC and SCC of the uterine cervix and molecular mechanisms of these differentially expressed genes have been suggested to contribute different clinical features of SCC and AC of cervix [20-22].

A number of putative co-factors such as tobacco smoke, inflammation and co-infections with other pathogens such as chlamydia and Herpes simplex virus contributing to HPV-induced cervical carcinogenesis have been identified by epidemiological case studies [51]. NO was reported to be one of the molecular co-factor, inducing early viral transcription in HPV infected cells [51]. The role of NO is also highly varied in cancer and several studies contradicting its role in progression of cancer had been reported [29]. Studies showing NO suppressing growth of ovarian tumor cells and human renal carcinoma cells have been reported [29]. Strong expression of iNOS and eNOS were found in breast cancers, intestinal cancers and other cancers [29]. The PI3K/AKT pathway is known to directly regulate NOS and production of NO [30, 31]. The role of DEPTOR in regulation of NOS enzymes through PI3K/AKT was documented in this study (Figure 2A and 2B; Figure 3A and 3B), hinting the importance of DEPTOR in various signaling mechanism. The role of NOS enzymes and its regulation by DEPTOR still needs to be studied in-detail to ascertain its importance in cervical carcinogenesis.

PI3K-AKT pathway is the central downstream effector of several growth factor receptors and its de-regulation was highly documented in cancer. Activation of PI3K-AKT pathway leads to the activation of several downstream molecules that attribute strong proliferative, anti-apoptotic and metabolic effects of the pathway [34]. HPV 16 is known to activate the PI3K/AKT/mTOR pathway [15]. In the present study, we show that DEPTOR silencing activated S6K and 4E-BP1 via mTORC1, thereby imparting a strong negative feedback inhibition on IRS1-PI3K-AKT in both SiHa and ME-180 cells (Figure 3A and 3B). Feedback inhibition of AKT signaling was known to limit the growth of tumors, hinting at the proper balancing of PI3K-AKT by S6K in cells under normal physiology, which when disturbed has serious implications in metabolic diseases and tumorigenesis [4, 52-54]. DEPTOR silencing in SCC cells resulted in the up-regulation of p53 and PUMA (Figure 2A and 2B). AKT inhibits p53 tumor suppressor protein through Mdm2 by nuclear translocation of Mdm2 and diminishes cellular p53 levels and its transcriptional activity, and pharmacological blockade of PI3K/AKT pathway increases activity of p53 [34]. The reduced AKT activity as a result of DEPTOR silencing could account for the elevated p53 and PUMA proteins, contributing to apoptosis.

The role of ERK and p38 MAP kinases was always found to be varied when it comes to cell survival and apoptosis. Although DEPTOR binds to mTOR endogenously, it was also observed that DEPTOR contains a putative ERK binding domain [55] and also associated with ERK in cells that have been co-transfected with a Flag-tagged DEPTOR construct [50]. In the current study, DEPTOR silencing in SCC cells resulted in the up-regulation of both ERK and p38 MAPK (Figure 6A and 6B). The up-regulated forms of ERK1/2 and p38 MAPK might have also contributed to the apoptosis of cells under DEPTOR silencing. The role of ERK in promoting apoptosis under DEPTOR silencing was investigated in this study and the results indicate, ERK was not found to promote the apoptosis in DEPTOR silenced cells, as cells pre-treated with ERK inhibitor did not overcome the cell death caused by DEPTOR silencing. In our study, direct interaction between endogenous DEPTOR and ERK1/2 was not observed, however the clear activation of ERK and p38 MAPK by DEPTOR silencing suggests a positive input by mTORC1 activation. While there are reports citing that activated PI3K/AKT directly activates MAPK [56], the present study reveals that inhibition of PI3K/AKT, activates ERK. The suppression of ERK1/2 activity under DEPTOR silencing conditions in cells overexpressing CA-AKT (Figure 5B) also supports this statement.

The role of p38 MAPK in promoting apoptosis under DEPTOR silencing was also elucidated. The results clearly indicate that p38 MAP kinase is responsible for the apoptosis under DEPOTR silencing, as cells pre-treated with p38 MAPK inhibitor attenuated the cell death caused by DEPTOR silencing (Figure 6B). Several reports indicate the strong role of p38 MAPK in the activation of p53 and further p53 mediated apoptosis [46, 47]. Previously reports also indicated that p38 MAPK pathway stabilizes the p53 protein level and this p38MAPK/p53 cascade regulates mesodermal differentiation and neurogenesis of embryonic stem cells [57]. All these reveal the role of p38 MAPK in the direct regulation of p53 and also p38 MAPK's role in promoting p53 mediated apoptosis. The strong up-regulation/activity of p38 MAPK under DEPTOR silencing strongly supports this. To our knowledge, this is the first study reporting the regulation of p38MAPK by DEPTOR. All these findings point towards the importance of DEPTOR in regulating various survival and death signaling, indicating its complex role (Figure 7D). The pathway cartoon (Figure 7D) outlines the possibilities of DEPTOR signaling elucidated in this study.

We hypothesized the possibility of interaction between HPV E6 and DEPTOR mediated by their PDZ domains, to sustain continuous activation of mTOR. Our repeated immunoprecipitation experiments however failed to reveal any possible interaction between DEPTOR and HPV E6 and also with HPV E7. DEPTOR silencing in HPV-negative cervical cancer cell C33A likewise induced a strong apoptotic response similar to SiHa and ME-180 cells and HPV E6/E7 silencing in SiHa also showed no difference in DEPTOR protein levels when compared to the control silenced cells (Figure 7). A similar study on NEDD9, a focal adhesion scaffolding protein, proposed to promote migration and invasion of cervical cancer was found to be independent of any interaction with HPV E6/E7 [58]. DEPTOR overexpression was observed in the primary SCC samples examined by IHC (Figure 8) and these studies needs to be applied to a large sample size to ascertain DEPTOR as a cytological marker in cervical SCC, along with already well-established markers like p16, cyclins such as B1 and E, and cell cycle marker Ki-67. DEPTOR was found to be strongly overexpressed in all the samples, with differential localization, when compared to the apparently normal cervical tissue (Figure 8; Supplementary Figure 4). DEPTOR was found to localize to cytoplasm, nucleus and cell membrane as revealed by IHC (Figure 8; Supplementary Figure 4). The mechanism of this localization of DEPTOR and its role in cellular processes needs to be elucidated further. Considering that HPV infection is an early event in the establishment of cervical cancer and as DEPTOR is not directly regulated by HPV oncoproteins E6/E7, we therefore hypothesize that action of DEPTOR to be a late molecular event in the pathogenesis or progression of cervical SCC, possibly beyond the severe dysplasia stages leading to carcinoma *in situ*.

### Conclusion

To conclude, DEPTOR is overexpressed in cervical SCC cells. DEPTOR overexpression in these cells is necessary for the sustained activation of PI3K/AKT activity needed for survival and proliferation of SCC cells. Our data implicate a strong feedback inhibition being relieved for a sustained PI3K-AKT activation by DEPTOR under natural conditions for SCC cell survival and proliferation. The present study also revealed the vital role of DEPTOR in complex molecular signaling involving PI3K/mTOR/AKT, ERK and p38 MAP kinases in cervical SCC cells. These results suggest not only a mechanism involving DEPTOR-mediated oncogenic signaling but also the rationale for therapeutic strategies halting progression of cervical pre-cancers. In-depth studies on the role of DEPTOR localization and its differential regulation in cancers further needs to be studied in detail. This study yet again points at the need of a comparative look at the transcriptional and translational levels between cervical SCC and AC, in approaching a better understanding of these cancers.

## MATERIALS AND METHODS

### Cell cultures

Cervical cancer cells SiHa, ME-180, HeLa and C33A were purchased from ATCC and resuscitated from earlier passage liquid nitrogen frozen stocks. All the cell lines were cultured in Dulbecco's Modified Eagle's Medium (DMEM) supplemented with 10% fetal bovine serum (FBS) (Pan life sciences, USA) and 1% penicillin/streptomycin (Gibco, USA) at 37°C and 5% CO_2_ and all the cells were routinely inspected microscopically for stable phenotype.

### siRNA and cDNA transfection

Knockdown of DEPTOR in cervical cancer cells were performed with 90 nM predesigned DEPTOR siRNA (DEPTOR siRNA is a 3 target specific pooled siRNA, sc-77660h, Santa Cruz, USA). A scrambled siRNA (control siRNA) duplex was used as a nonspecific negative control for silencing (Dharmacon, USA). In brief, SiHa and ME-180 cells were seeded into 6 well plates and DEPTOR knockdown was performed using Xtreme gene siRNA transfection reagent (Roche, USA). After 48 hours of transfection, cell lysates were prepared to assess the effects of DEPTOR silencing. For some experiments, constitutively active AKT (CA-AKT) ((pLNCX AKT CA was gifted by Dr. Tohru Minamino, Chiba University Graduate School of Medicine, Japan) was transfected in SiHa cells for its overexpression and further DEPTOR silencing was performed in these cells. DEPTOR overexpression studies were also performed in SiHa using Flag-DEPTOR plasmid (Gift from Dr. David Sabatini, MIT; Addgene plasmid # 21334). HPV E6/E7 siRNA [59] was also used as described above to assess their role in regulation of DEPTOR.

### Inhibitors and drug treatments

Rapamycin (Sigma, Germany) and Torin 2 (Tocris Biosciences, USA) inhibitors were used for the treatment of SiHa and ME-180 cells to assess their effects on signaling in comparison with DEPTOR silencing. The concentrations of rapamycin used (100, 200 and 500 nM) were as previously described [60]. Torin 2 is a potent inhibitor of both mTOR complexes [61]. The optimum cytotoxic concentrations of Torin2 that inhibit cell growth were determined for both SiHa and ME-180 cells by MTT assay to determine appropriate concentrations of Torin2 to inhibit cell growth.

SB202190 and PD98059 (Sigma, Germany) were used as p38 MAPK and ERK inhibitors for delineating their respective roles in apoptosis induced by DEPTOR silencing. 10 μM of both drugs were employed to inhibit the activity of respective proteins [62, 63].

### MTT assay

Cytotoxicity studies of Torin 2 were carried out in both SiHa and ME-180 cells (Supplementary Figure 1) to detect the appropriate concentration of the drug using MTT assay [64].

### Immunoblotting

Briefly, cells were washed with ice cold PBS and lysed with ice-cold phospholysis buffer supplemented with protease and phosphatase inhibitors. After lysis, the suspension was centrifuged at 12000 rpm for 10 minutes at 4°C, and the supernatant was collected and stored for further processing. The total protein content of the extracted whole cell lysates were determined by the Bradford method, and 50μg of the total extracted protein from respective samples were denatured and subjected to immunoblotting. The following primary antibodies and their respective secondary antibodies labeled with HRP were used for detecting the expression levels of various proteins with enhanced chemiluminescence (Pierce ECL substrate, USA). Primary antibodies used were DEPTOR, eNOS, iNOS, HPV E6, HPV E7, HSC-70 (Santa Cruz, USA); mTOR, phosphor-mTOR (ser 2448), phospho-pERK1/2, phospho-p38, phospho-4EBP1, phospho-S6K, AKT, phospho-AKT, PARP, Caspase 3, Caspase 9, Caspase 7, PUMA, p53 (Cell Signaling Technology, USA); pRb (Oncogene, USA); phospho-IRS1, phospho-GSK3β (Pierce, USA); Vinculin, anti-rabbit HRP, anti-goat HRP, anti-mouse HRP antibodies (Sigma, Germany).

### Annexin V assay for quantification of apoptosis using FACS

Translocation of phosphatidylserine (PS) from the inner to extracellular membrane leaflet, thus exposing PS to the external environment, is an important marker of early apoptosis that can be detected by annexin V conjugate. Briefly, cells were harvested, centrifuged and again washed with ice cold PBS. The cell pellet was re-suspended in the annexin binding buffer, probed with annexin V conjugate (Sigma, Germany) and further analyzed by FACS (FACS Aria-I, BD Biosciences, USA) for the detection of annexin positive cells.

### Nuclear condensation assay

Cells were seeded in 96 well plates and DEPTOR silencing was performed in both SiHa and ME-180 cells with respective scramble siRNA silencing. After 36 hours of silencing, the cells were stained with Hoechst (0.5 μg/ml) for 10 minutes and washed with PBS. Cells were imaged for nuclear condensation using fluorescence microscopy (Nikon, Japan).

### Confocal microscopy

SiHa cells stably expressing cytochrome C fused with EGFP were seeded in 96 well plate and incubated at 37°C with 5% CO_2_. DEPTOR silencing was performed as described above. After 36 hours, the media was changed and the cells were imaged by confocal microscopy (Nikon, Japan) for Cyt C release, which is an event during apoptotic induction [65].

### Colony formation assay

Clonogenic assay was performed using SiHa cells as described by Franken et al., [66].

### Immunohistochemistry

Formalin-fixed, paraffin-embedded (FFPE) tissue sections of 4-μm thickness were taken from histologically confirmed Invasive cervical squamous cell carcinoma and from normal cervical tissue. The studies were approved by the Human Ethics Committee. Sections were deparaffinized and then rehydrated serially with two changes of xylene and descending grades of isopropanol respectively. All slides were subjected to heat-induced epitope retrieval using sodium citrate buffer. Endogenous peroxidase activity in tissues was blocked by incubation with 6% hydrogen peroxide solution for 30 minutes. The slides were stained for DEPTOR with a 1:100 dilution of anti-human DEPTOR antibody, and signal visualization by diaminobenzidine (SS polymer-HRP-DAB detection kit-BioGenex, USA). Stained slides were counterstained with haematoxylin and DEPTOR expression was evaluated based on the intensity of staining.

## SUPPLEMENTARY FIGURES



## Abbreviations

DEPTOR: DEP domain containing mTOR inhibitor
HPV: Human papillomavirus
SCC: Squamous cell carcinoma
AC: Adenocarcinoma
NOS: Nitric oxide synthase
iNOS: inducible Nitric oxide synthase
eNOS: enodothelial Nitric oxide synthase
Cyt C: Cytochrome C
siRNA: Small interfering RNA
