# Supplementary material for: DEPTOR promotes survival of cervical squamous cell carcinoma cells and its silencing induces apoptosis through downregulating PI3K/AKT and by up-regulating p38 MAP kinase
Source: Oncotarget. 2016 Mar 16;7(17):24154–71. doi: 10.18632/oncotarget.8131 (PMC5029691; doi:10.18632/oncotarget.8131)
Supplement: Supplementary file 1 [file oncotarget-07-24154-s001.pdf]

SUPPLEMENTARY FIGURES

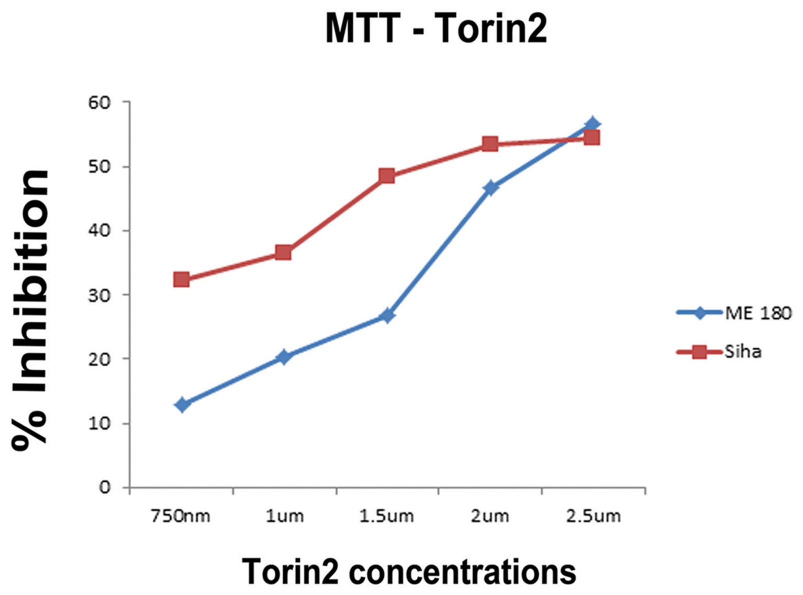

Supplementary Figure S1: MTT assay for Torin2.

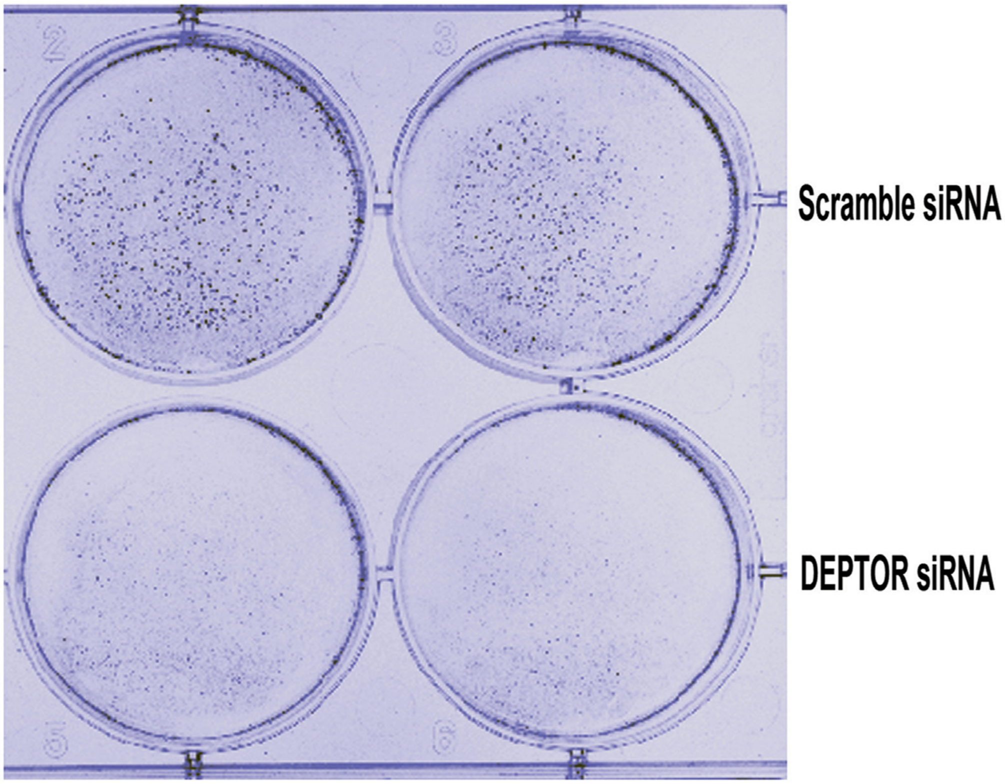

Supplementary Figure S2: Clonogenic assay of DEPTOR silenced SiHa cells.

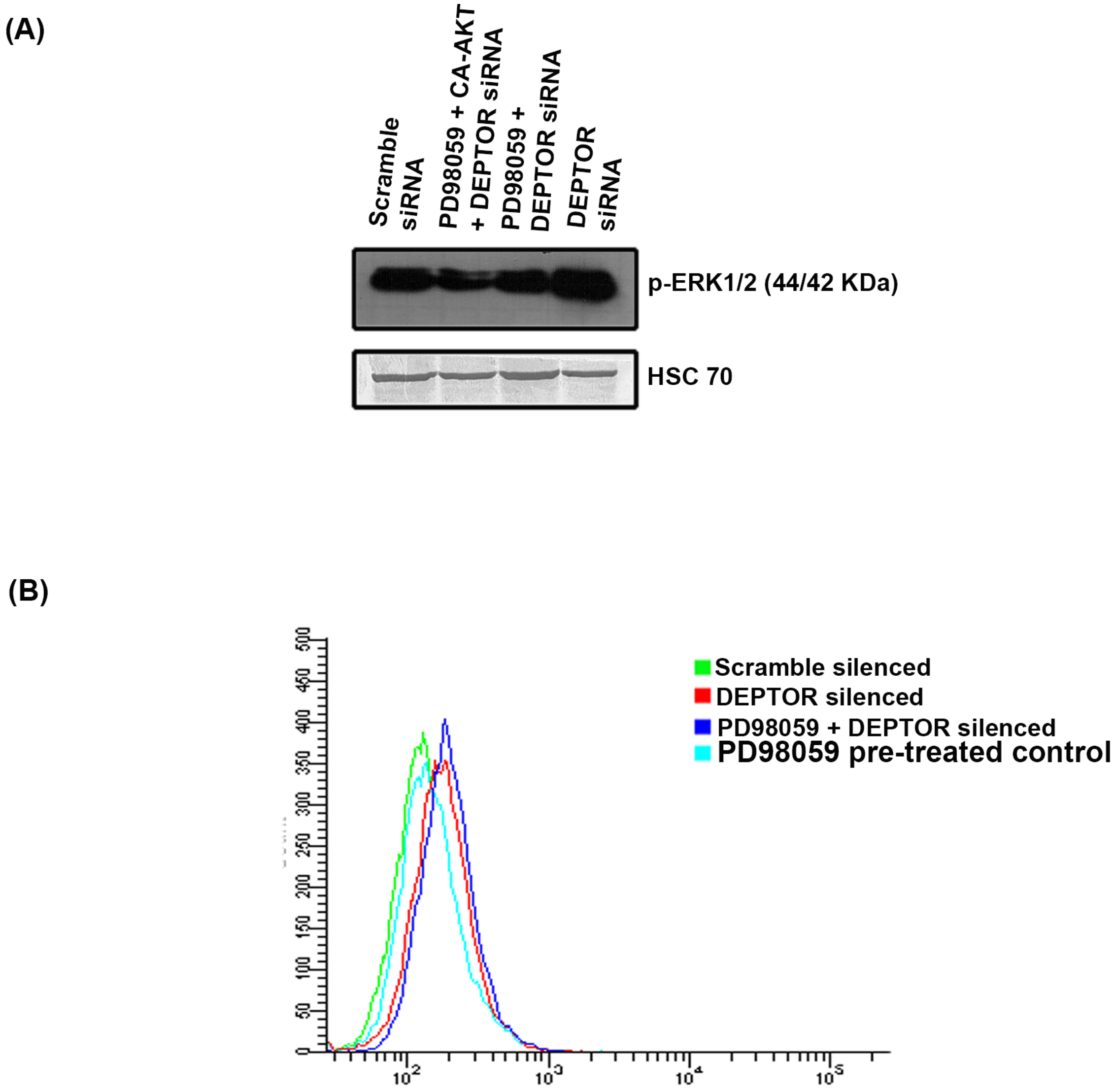

Supplementary Figure S3: DEPTOR silencing in cells pretreated with ERK inhibitor. A. Western blotting shwing status of p-ERK1/2 under DEPTOR silencing with ERK inhibitor PD98059 pretreatment (10 uM) and CA-AKt overexpressed conditions. **B.** Annexin binding assay using FACS indiactes strong annexin positivity/apoptotic induction in cells pretreated with ERK inhibitor and DEPTOR silencing, similar to DEPTOR silencing alone in comparison to respective controls.

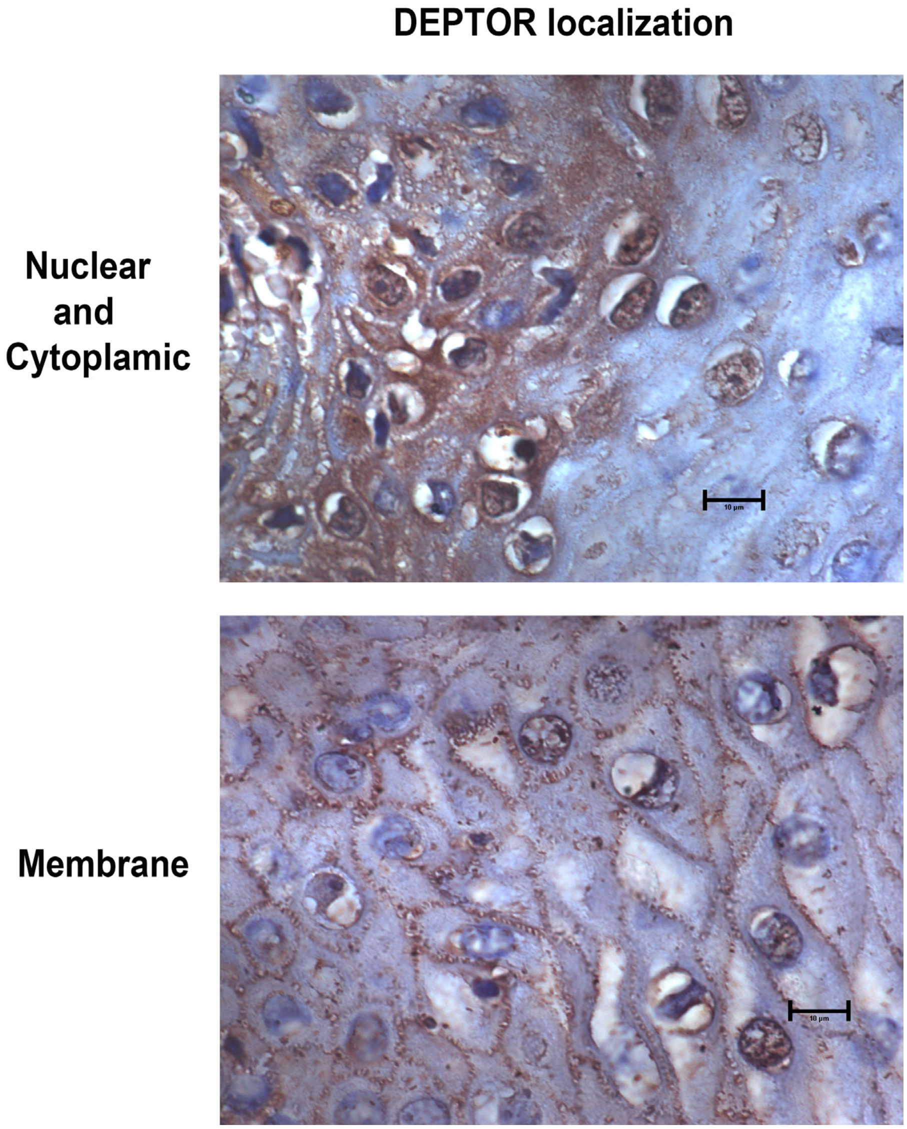

Supplementary Figure S4: DEPTOR localization.
